# Pressure pain mapping of equine distal joints: feasibility and reliability

**DOI:** 10.3389/fpain.2024.1342954

**Published:** 2024-04-25

**Authors:** Jana Gisler, Ludovica Chiavaccini, Severin Blum, Stéphane Montavon, Claudia Spadavecchia

**Affiliations:** ^1^ Department of Clinical Veterinary Medicine, Anaesthesiology and Pain Therapy Section, Vetsuisse Faculty, University of Bern, Bern, Switzerland; ^2^ Veterinary Department of the Swiss Armed Forces, Bern, Switzerland; ^3^ Department of Comparative, Diagnostic, and Population Medicine, College of Veterinary Medicine, University of Florida, Gainesville, FL, United States

**Keywords:** pain, equine, osteoarthritis, pressure algometry, complementary diagnostic

## Abstract

**Background:**

Osteoarthritis is a prevalent degenerative joint disease initiating chronic pain and lameness in horses. While several objective gait analysis systems have been developed and validated to quantify lameness severity in horses, methods to evaluate whether peripheral sensitization contributes to the pain experienced are missing.

**Objectives:**

To evaluate whether periarticular pressure pain mapping could be proposed as an auxiliary assessment tool in horses. Specific aims were to evaluate the feasibility and intra- and inter-rater reliability of pressure pain thresholds (PPT) determination at sites overlying the distal thoracic limb joints of clinically healthy horses.

**Study design:**

Prospective, randomized validation study.

**Methods:**

For feasibility assessment, PPT were measured with a hand-held digital algometer at six periarticular landmarks (2 sites per joint, 3 joints) bilaterally on the distal thoracic limb of 40 healthy horses (20 warmblood and 20 Freiberger). The joints tested were the metacarpophalangeal, on the latero-palmar and dorsal aspects (L-MCP and D-MCP), the proximal interphalangeal, on the dorsal and palmar aspect (D-PIP and P-PIP) and the distal interphalangeal, on the dorsal and lateral aspect (D-DIP and L-DIP). A feasibility score, ranging from 0 to 5, was attributed to each testing session. For intra- and inter-rater reliability assessment, L-MCP and D-MCP were selected to be tested again at 2 weeks intervals in 20 out of the 40 horses. Data were analyzed using a mixed-effect linear model to test differences in threshold per site and limb. Intra- and inter-rater correlation was calculated. Bland-Altman plots were performed to evaluate the variability of the measures.

**Results:**

The procedure was considered feasible (score <2) in 95% of horses (95% CI 88%–100%). Overall, median [interquartile range (IQR)] PPT was 9.4 (7.5–11.3) *N*. No significant side differences were found. P-PIP and D-DIP recorded significantly lower PPT (*p* < 0.001 and *p* = 0.002, respectively) than L-MCP. Median (IQR) were 9.9 (7.3–12.4) *N*, 8.4 (6.1–10.5) *N* and 9.0 (7.4–10.6) *N* for L-MCP, P-PIP and D-DIP, respectively. The intra-rater agreement was 0.68 (95% CI 0.35–0.86) for L-MCP, and 0.50 (95% CI 0.08–0.76*)* for D-MCP. Inter-rater agreement was 0.85 (95% CI 0.66–0.94*)* for L-MCP and 0.81 (0.57, 0.92) for D-MCP.

**Main limitations:**

Evaluation of feasibility was performed only for distal thoracic limbs joints; no data are provided for hind limbs or proximal joints. Only warmblood and Freiberger horses were included. Intra- and inter-rater reliability assessments were performed exclusively on data collected at the MCP joint.

**Conclusion:**

Pressure pain mapping of distal thoracic limb joints was feasible in horses. Local sensitivity differed among sites and no side differences were noticed. Data collected from the MCP joint suggest highly variable, subject dependent intra-rater reliability, ranging from poor to good, and good to excellent inter-rater reliability. Further studies evaluating pathologic vs. healthy joints are needed before recommendations can be made about clinical usability and diagnostic validity.

## 1 Introduction

Osteoarthritis (OA) is a degenerative joint disease prevalent in horses as well as in many other species, including dogs, cats and humans. The predominant symptoms are chronic pain and lameness. Although the latter is the chief reason for impaired athletic performance and quality of life of affected horses, the mechanisms behind joint pain in OA are still poorly understood. Osteophytes and periosteal elevation, cartilage abnormalities, subchondral cysts, increased intraosseous pressure in the subchondral bone, bone marrow lesions and inflammation of the synovial membrane have all been reported to be potential local contributors to the generation of chronic pain accompanying OA (1, 2). However, strikingly, typical OA radiographic changes are only weak risk factors for the occurrence of pain and the severity of structural changes is not necessarily associated with pain intensity in both horses (3) and humans (4, 5). In contrast, the impairment of autonomic joint innervation, the plasticity of nociceptive fibers supplying periarticular structures, as well as the sensitization and dysfunction of descending pain inhibition mechanisms might be crucial promoters of pain development and maintenance in OA (2, 6). Which factor or factor combination predominates in individual patients remains to be determined.

While several objective gait analysis systems have been developed and validated to quantify lameness severity in horses (7–9), methods to evaluate whether simultaneous peripheral and central sensitization mechanisms are contributing to the pain experienced are missing. In humans, recently developed advanced and thorough sensory testing methods allow the characterization of the patient-specific OA pain phenotype (10, 11). For example, pressure pain sensitivity maps projected on tridimensional contour models can be constructed for individual patients and specific joints to obtain a visual impression of sensitivity distribution; similarly, using computer controlled mechanical stimulation, temporal summation can be assessed by repeating subthreshold stimulations and determining the extent of facilitation, which might reflect the presence of central sensitization. Such a personalized approach promotes mechanism-based therapy and, thus, better pain relief with fewer adverse effects (12). A validated, reliable and quantitative method to assess periarticular sensitivity in OA-affected horses is essential to establish the extent of peripheral sensitization involvement and, thus, to provide adequate and individualized treatment.

In humans and dogs, the non-invasive technique of pressure pain mapping, also known as pressure algometry or mechanical nociceptive threshold testing, has been used to quantify sensitization in several musculoskeletal conditions, including OA (13–16). So far, pressure pain mapping has not been used to quantify naturally occurring OA-associated pain in horses. At the same time, it has been described to assess limb sensitization in a model of experimentally induced carpal OA (17). Several trials explored the usability of pressure algometry to evaluate back pain (18, 19) or alteration of limb sensitivity (20, 21) in equines, and a summarizing review of results obtained with this method in horses has been recently published (22). On the other hand, the feasibility and reliability of periarticular pressure pain mapping have never been evaluated in horses.

The study objectives were to evaluate the feasibility and intra- and inter-rater reliability of PPT measured bilaterally over the distal thoracic limb joints in healthy horses. It was hypothesized that: (1) pressure pain mapping of the distal equine joints would be feasible; (2) repeatability and reliability of PPT mapping would be overall acceptable.

## 2 Materials and methods

The study was approved by the Committee for Animal Experimentation of the Canton of Bern, Switzerland (license number BE81/2022). The trial was carried out at the National Equine Center in Bern from October 2022 to January 2023.

### 2.1 Horses

Forty healthy Swiss warmblood (*n* = 20) and Freiberger (*n* = 20) horses, mare and geldings, aged >3 years and belonging to the Swiss Armed Forces were included. Horses were kept in single stalls in large stables under standard housing conditions and were regularly ridden or driven. Prior to study inclusion, a complete physical examination was performed by army veterinarians (JG, SB) supervised by an experienced equine specialist (SM) (Supplementary Material Appendix S1 for details). Lameness was assessed using the Americal Association of Equine Practitioners (AAEP) scale (0–5, with 0 indicating a normal gait and 5 non-weight bearing lameness) on a hard, straight surface at walk and trot. Distal thoracic limb joints were visually observed and palpated. The degree of joint effusion, reaction to palpation and reaction to flexion were subjectively scored using a numerical rating scale (0 = absent; 1 = mild; 2 = moderate; 3 = severe).

To be included, horses had to be free of clinically detectable orthopedic, neurologic or systemic diseases and have no evidence of pain or mobility impairment. Lameness, joint effusion, reaction to palpation and to flexion had to be ≤1. Horses were excluded if they received any anti-inflammatory or analgesic drugs in the 2 weeks prior to the study. Physical and orthopedic examinations were repeated before each experimental session to ensure that no changes had occurred between appointments. If lameness or other symptoms appeared, horses were excluded from further testing, and the noticed clinical issue was recorded. Skinfold thickness was measured with a caliper (Universal Vernier Caliper, Tesa, Switzerland) over the scapula and cranial to it at the neck basis, anticipating that this factor might influence potential breed differences in PPT. Time from the last shoeing was recorded; no experiment was performed during the first week following shoeing. All horses were tested in the afternoon, in their own stall, manually held by the halter and lead rope by an assistant not performing the measurements. At least 1 h had to elapse between feeding or daily training exercise and testing.

### 2.2 Study design

Three consecutive study phases were designed to reach the set goals.

#### 2.2.1 Phase 1

This phase aimed at assessing feasibility, site sensitivity and left-right differences of distal limb joints PPT mapping.

Horses were acclimated with the stimulation method with an initial short training period just preceding the beginning of the experiment. During this period, the algometer was applied to at least three undefined sites distal to the metacarpophalangeal joint, not corresponding to the test sites, until the horse showed a response. Thereafter, the actual experiment began.

**Figure 1:**
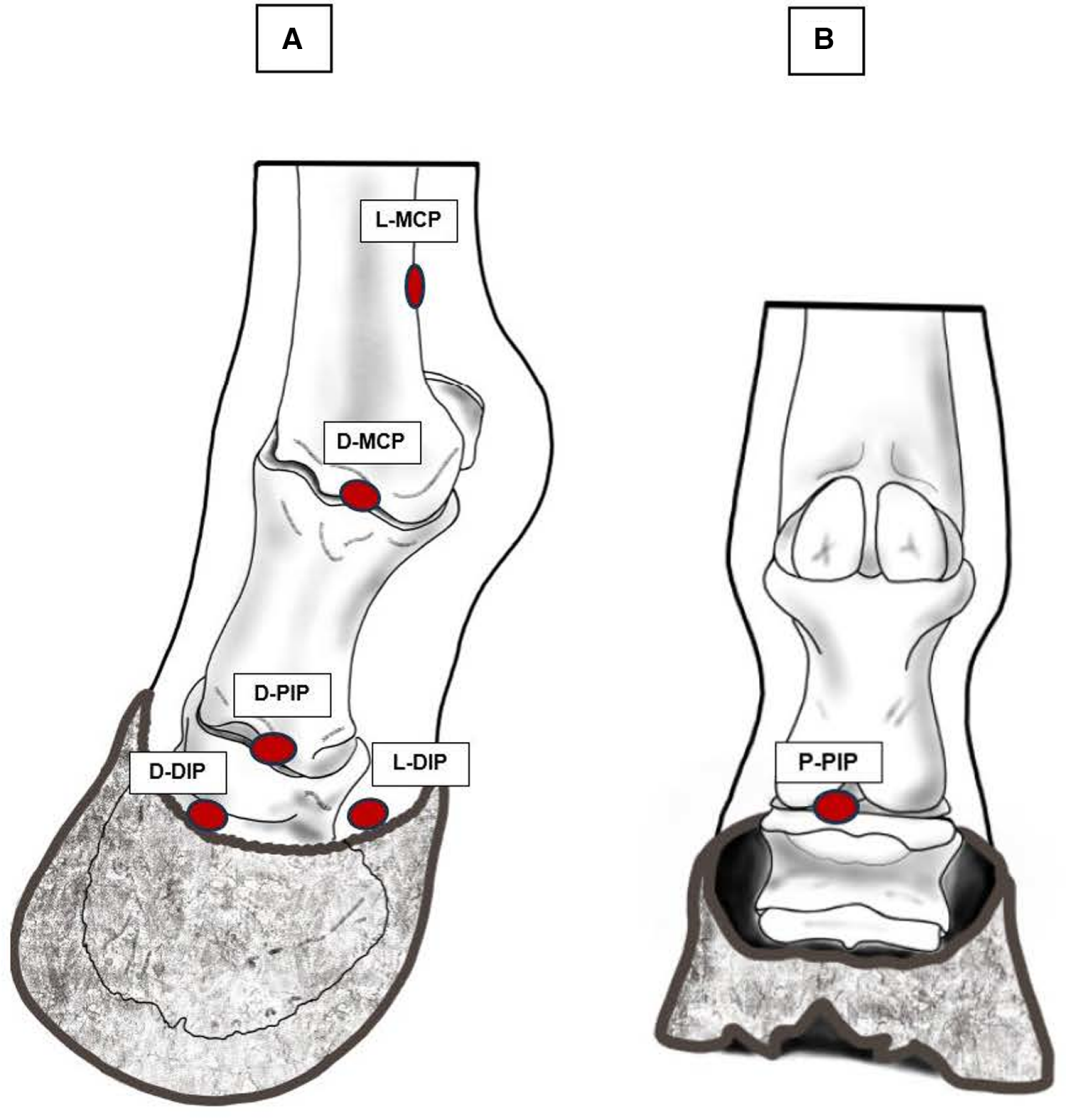
The 6 periarticular sites tested with algometry. (**A**) Dorso-lateral view of the distal thoracic limb, L-MCP and D-MCP on the latero-proximal-palmar and dorsal aspect of the metacarpophalangeal joint, respectively, D-PIP on the dorsal aspect of the proximal interphalangeal joint, D-DIP and L-DIP on the dorsal and lateral aspect of the distal interphalangeal joint, respectively. (**B**) Palmar view of the distal thoracic limb, P-PIP, on the palmar aspect of the proximal interphalangeal joint. Original drawings inspired by images available in reference (23).

The same observer (JG) performed all the PPT measurements in this phase. All 40 horses were tested bilaterally at six sites, two per joints (Table 1 for anatomical details). The sites were the L-MCP and D-MCP, on the latero-proximal and dorsal aspect of the metacarpophalangeal joint, the D-PIP and P-PIP, on the dorsal and palmar aspect of the proximal interphalangeal joint, and the D-DIP and L-DIP, on the dorsal and lateral aspect of the distal interphalangeal joint (Figure 1). These sites correspond to those described to perform arthrocentesis in the same joints (23).

**Table 1:** Periarticular stimulation sites.

Sites	Description
L-MCP	Metacarpophalangeal joint, latero-proximal-palmar aspect, midway between the distal end of the fourth metacarpal bone and the lateral proximal sesamoid bone
D-MCP	Metacarpophalangeal joint, dorsal aspect, lateral to the common digital extensor tendon at the level of the palpable joint space
D-PIP	Proximal interphalangeal joint, dorsal aspect, distal to the lateral bony eminence of the proximal phalanx and lateral to the common digital extensor tendon
P-PIP	Proximal interphalangeal joint, palmar aspect, proximal and central to the transverse bony prominence of the middle phalanx
D-DIP	Distal interphalangeal joint, dorsal aspect, central above the coronary band
L-DIP	Distal interphalangeal joint, lateral aspect, proximal to the lateral collateral cartilage, midway between the dorsal and palmar sides of the middle phalanx

#### 2.2.2 Phase 2

This phase aimed to assess intra-rater reliability for a single observer (JG). Twenty horses randomly selected from the initial cohort were tested at least 2 weeks after Phase 1. Horses who recorded a feasibility score >2, were removed from the pool before randomization. The two sites on the metacarpophalangeal joint, L-MCP and D-MCP, were evaluated on the left and right thoracic limb. Data collected during Phase 2 were then compared to those collected in Phase 3 by the same observer.

#### 2.2.3 Phase 3

This last phase aimed to assess intra-rater reliability and generate the data set to evaluate inter-rater reliability. Two observers (JG and SB) performed PPT testing during this session.

The same horses included in Phase 2 were included in Phase 3. At least 2 weeks elapsed between Phases 2 and 3. Both observers evaluated the horses once; they tested the two metacarpophalangeal joint sites (L-MCP and D-MCP) on both thoracic limbs in the same order, the first observer being randomized for each horse with the flip of a coin. At least 60 min elapsed between observers.

### 2.3 Pressure pain threshold determination

For PPT determination, a hand-held digital algometer, equipped with a flat 2 mm diameter tip (ProdPro, Top Cat Metrology Ltd, UK), was applied perpendicular to the skin at predefined periarticular sites (Table 1 for anatomical details) until a behavioral reaction was elicited. A constant force rate increase of 2 N/s was kept with the guidance of warning LED lights on the instrument. During stimulation, the operator was not aware of the applied force. Stimulation was stopped when a weight shifting to the contralateral limb, a voluntary limb lifting and/or stamping occurred, or when the cut-off force of 25 N was reached. At this point, the operator withdrew the probe, and the peak force (N) displayed on the device was recorded as the threshold.

The limb (left or right) and the order of sites to be tested were randomized for each horse with the flip of a coin and a random number generator (https://www.matheretter.de/rechner/zufallsgenerator), respectively. Each site was tested three times and the two closest values were averaged for further analysis. The same order of site testing was kept for each repetition during a session. Minimal inter-stimulation interval was 20 s, with 60 s between tests on the same site. If the horse moved or the algometer jerked or slipped, the measurement was repeated.

During the whole stimulation session, particular care was dedicated to ensuring a quiet environment and keeping the general behavior of the horse under strict observation. If the horse appeared distracted from noises or other external conditions during threshold assessment, the stimulation was interrupted and repeated once the horse returned to a calm, concentrated attitude.

Complete physical and orthopedic examinations were repeated 1 day and 1 week after each experimental session for safety monitoring. Furthermore, tested joints and periarticular skin areas were regularly inspected for the presence of abnormal sensitivity to touch, temperature, swelling, lesions, or any other abnormality that could be noticed.

### 2.4 Feasibility assessment

At the end of each session, each horse was assigned a feasibility score that described the ease with which data could be collected (Table 2), adapting a scoring system previously described for dogs (24). Feasibility was scored on a scale ranging from 0 to 5, where scores ≤2 represented “easy data collection” and those >2 “difficult data collection”. Horses scoring >2 were excluded from subsequent testing to avoid unnecessary discomfort.

**Table 2:** Feasibility scores for evaluation or the ease with which pressure pain thresholds data were collected.

Score	Description
0	No problem. Minimum restraint needed; excellent cooperation; clear reaction to stimuli
1	Mild difficulty. Mild restraint needed; good cooperation; clear reaction to stimuli
2	Moderate difficulty. Moderate restraint needed; good cooperation >50% of the time; mild sensitivity of extremities being touched; mild variation in reaction to stimuli
3	Significant difficulty. Significant restraint needed; good cooperation <25% of the time; moderate sensitivity of extremities being touched; moderate variation in reaction to stimuli
4	Extreme difficulty. Constant restraint required; not cooperative; unclear reaction to stimuli, not confident in data collected
5	Impossible. Could not collect data due to the horse's disposition and/or lack of confidence in the reactions seen being due to the stimulus

Modified from (24).

### 2.5 Sample size

Sample size was calculated using a web-based tool based on previously described methods (25, 26). Assuming an ICC *ρ* = 0.6, with an expected width of the 95% confidence interval (CI) of 0.4, two PPT values per subject and a drop-out rate of 10%, 13 horses were considered necessary to assess reliability. As for reliability testing a group of 20 subjects was suggested to be a clinically representative sample (27), the number of horses included in the present study was increased to 20 per breed in Phase 1 and to 20 in total in Phases 2 and 3.

### 2.6 Statistical analysis

Data elaboration and analysis were performed with Stata/BE 17.0 for Mac (StataCorp LLC, College Station, TX) and SigmaPlot 14 (Systat Software, Palo Alto, CA). Descriptive statistics was used for demographic data. Continuous data were checked for normality of distribution using the Shapiro-Wilk normality test and graphically with histogram and the normal quantile plot function in Stata. Since the normality assumption was not met, data were reported as median [interquartile range (IQR)]. Categorical data were presented as proportions or percentages. The Mann-Whitney Rank Sum test was used to compare skinfold thickness between breeds.

In order to consider PPT mapping suitable for clinical use, the horse needed to score ≤2 in the feasibility score. A one-sample proportion *z*-test was used to compare the observed proportion of the sample to the 70% cutoff proposed in clinical feasibility studies in other species (24).

In Phase 1, to check the effect of breed, testing site and side on PPT, a mixed effect linear model was used, with the horse as the random effect, breeds, the six sites tested and left and right sides as fixed effects. Due to a lack of normality distribution, PPT was transformed using the Box-Cox transformation prior to analysis. For Phases 2 and 3, the intra- and inter-class correlation coefficients (ICC) were calculated, and the 95% CI was assessed to identify the precision of the estimate. The ICC values were classified as follows: <0.20 indicated poor agreement; 0.21–0.40 fair agreement; 0.41–0.60 moderate agreement; 0.61–0.80 good agreement and >0.80 excellent agreement (28). Systematic error between sessions and between raters was estimated using the Wilcoxon signed-rank test. *P*-values ≤0.05 were considered statistically significant. Bland-Altman plots were performed to graphically represent differences between two consecutive PPT measurements and between the two raters.

## 3 Results

A total of 40 healthy horses were included in the study: 20 Warmblood and 20 Freiberger. The median (IQR) age of horses was 4 (3, 7) year-old and they weighed 540 (495, 570) kg. The sample included 14 (35%) mares and 26 (65%) geldings. There were 5 (25%) mares and 15 (75%) geldings in the Warmblood group, and 9 (45%) mares and 11 (55%) geldings in the Freiberger group (*p* = 0.19). Skinfold thickness was significantly different between breeds at both tested sites, being 4.5 (4.0–4.8) mm in the Warmblood group and 5 (4.5–5.5) mm in the Freiberger group at the scapula (*p* = 0.023), and 4 (4–4.5) mm in the Warmblood group and 4.8 (4–5) mm in the Freiberger group at the neck basis (*p* = 0.004).

### 3.1 Feasibility and site specificity

In Phase 1, only two horses received a feasibility score >2, because they were moving too much their thoracic limbs and were then excluded from Phases 2 and 3. Consequentially, the procedure was then deemed “feasible” in 95% of the animals (38 out of 40 horses), which was statistically significantly superior to the hypothesized 70% (95% CI 88%–100%, *p* < 0.001).

A total of 480 PPT observations were collected in Phase 1, ranging between 1.5 and 25 N [the median (IQR) PPT obtained was 9.4 (7.5, 11.3) N] (Table 3). The final multivariable regression model was significant and predicted the data well (Wald chi2 = 67.28, *p* < 0.001). When accounting for breed differences and for the side (left or right), P-PIP (*p* < 0.001) and D-DIP (*p* = 0.002) recorded a significantly lower mechanical threshold compared to L-MCP. Regardless of the side, the median (IQR) mechanical threshold was 8.4 (6.1, 10.5) N for P-PIP and 9.0 (7.4, 10.6) N for D-DIP compared to 9.9 (7.3, 12.4) N for L-MCP. There was no difference in mechanical thresholds between breeds (*p* = 0.14) or sides (*p* = 0.33). Given the lack of statistically significant difference of PPT obtained from the left and right sides at each site, the PPT values of the left and right sides were averaged for further analysis.

**Table 3:** Median (IQR) pressure pain thresholds in 40 healthy horses.

Site	PPT left limb (N)	PPT right limb (N)
L-MCP	9.9 (7.7, 11.3)	9.8 (7.3, 14.9)
D-MCP	9.6 (7.6, 12.2)	9.5 (7.4, 11.5)
D-PIP	10.3 (7.8, 12.1)	9.9 (8.1, 10.9)
P-PIP	9.0 (6.4, 12.3)	7.9 (6.0, 10.1)
D-DIP	9.0 (6.7, 10.9)	9.1 (7.4, 10.0)
L-DIP	9.3 (8.3, 12.3)	9.8 (8.2, 11.9)

Data at each site were obtained averaging the closest two of three consecutive measurements. All measurements were performed by one observer.

### 3.2 Intra-rater reliability

For L-MCP, the median (IQR) PPT obtained by the first observer (JG) in the first measurement was 10.4 (9.4, 11.8) N and in the second one, 2 weeks later, 9.8 (8.8, 12.9) N. For D-MCP, the median (IQR) PPT obtained was 10.1 (8.8, 12.6) N in the first measurement and 11.1 (9.2, 12.8) N in the second one. The intra-rater agreement was 0.68 (95% CI 0.35–0.86) for L-MCP, and 0.50 (95% CI 0.079–0.76) for D-MCP, indicating moderate-to-good and poor-to-good repeatability, respectively (Table 4). The median difference between the first and second measurements was 0.55 N (95% CI −0.98, 1. 85; *p* = 0.62) for L-MCP and −0.35 N (95% CI −1.97, 1.30; *p* = 0.34) for D-MCP, indicating lack of systematic error in measurements.

**Table 4:** Intra-rater agreement (ICC) and 95% confidence interval of pressure pain thresholds measurements made 2 weeks apart on 20 horses by one rater.

Intra-rater	ICC (95% CI)	Systematic error		
		Median difference	95% CI	*p*-value
L-MCP	0.68 (0.35–0.86)	0.55 N	−0.98, 1.85 N	0.62
D-MCP	0.50 (0.08–0.76)	−0.35 N	−1.97, 1.30 N	0.34

### 3.3 Inter-rater reliability

For L-MCP, the median (IQR) PPT recorded by rater 1 (JG) and 2 (SB) were 9.8 (8.8, 12.9) N and 11.2 (9.0, 13.0) N, respectively. For D-MCP, the median (IQR) PPT recorded by rater 1 and 2 were 11.1 (9.2, 12.8) N and 11.3 (9.6, 11.7) N, respectively. The agreement was good-to-excellent between raters for both sites, being 0.8 (0.7–0.9) for L-MCP and 0.8 (0.6–0.9) for D-MCP (Table 5). Median difference between raters was −0.61 N (95% CI −2.52, 1.42; *p* = 0.59) for L-MCP and 0.14 (95% CI −1.40, 1.45, *p* = 0.83) for D-MCP, indicating lack of systematic error in measurements.

**Table 5:** Inter-rater agreement (ICC) and 95% confidence interval of pressure pain thresholds measurements made on 20 horses by two raters, on the same day at 1 h interval.

Inter-rater	ICC (95% CI)	Systematic error		
		Median difference	95% CI	*p*-value
L-MCP	0.85 (0.66–0.94)	−0.61 N	−2.52, 1.42 N	0.59
D-MCP	0.81 (0.57–0.92)	0.14 N	−1.40, 1.45 N	0.83

**Figure 2:**
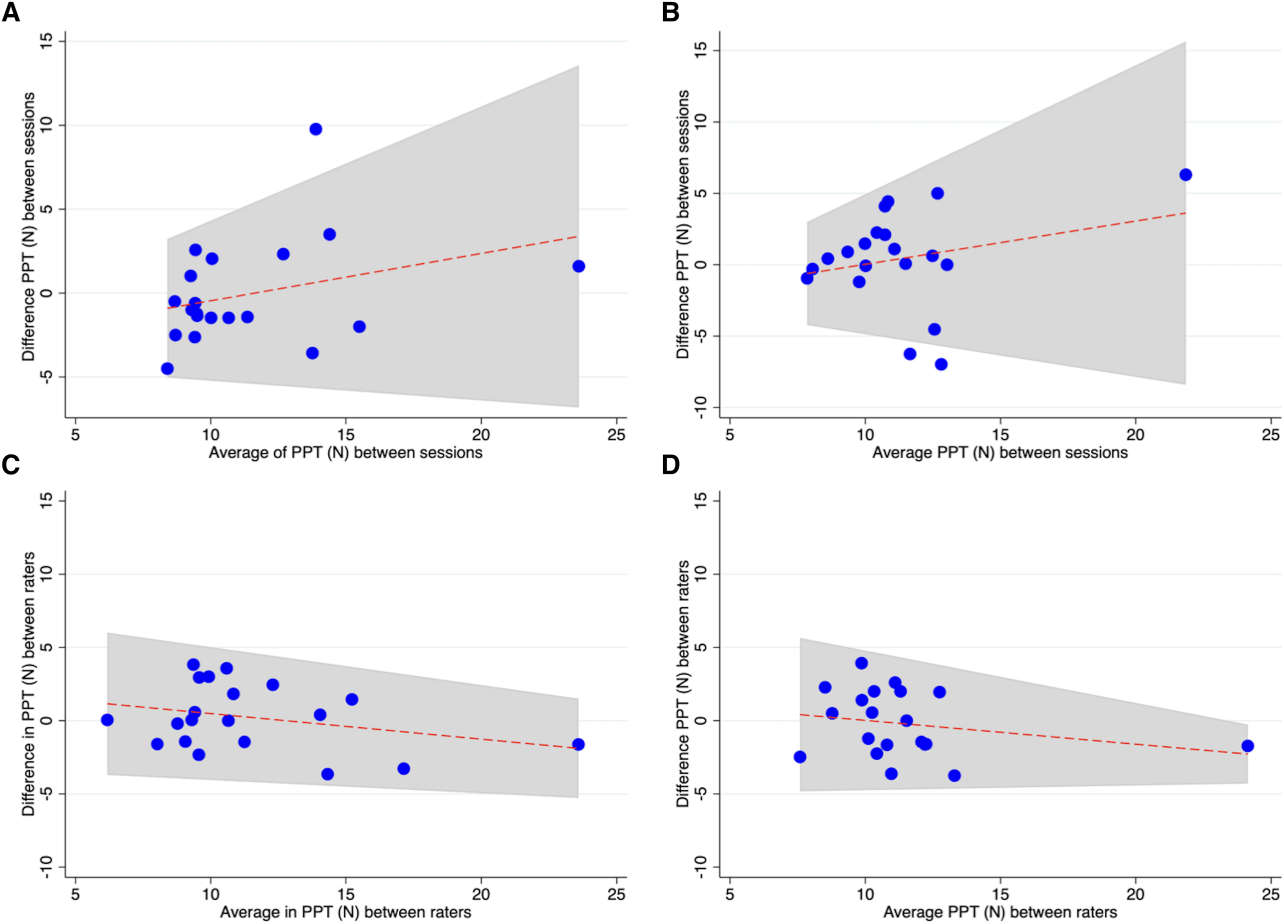
Bland-Altman plots shows distribution of PPT for each site and rater: (**A**, **B**) PPT recorded between two sessions from rater 1 at L-MCP and D-MCP, respectively. (**C**, **D**) PPT recorded between raters at L-MCP and D-MCP, respectively.

The Bland-Altman plots for all the evaluations are included in Figure 2 illustrating the distribution of the PPT values difference when plotted against the mean and estimate an agreement interval within which 95% of the differences lied.

No adverse events possibly related to the testing procedures were observed throughout the study.

## 4 Discussion

The main finding of this study was that periarticular PPT showed excellent feasibility when 6 periarticular sites were tested bilaterally on the distal thoracic limbs of clinically healthy horses. Tested side and breed did not affect thresholds. Reliability, evaluated on a single joint, was quite variable, being better when 2 different raters performed the tests on the same day than when the same sites were tested by the same observer 2 weeks apart. The overarching aim was to verify whether this quantitative sensory testing method, already used in humans and dogs affected by chronic joint pain (10, 14, 16), could be proposed to assess periarticular sensitivity in horses affected by OA. Indeed, the crucial role of peripheral sensitization and thus enhanced local sensitivity to the overall pain experience in OA has often been highlighted (1, 2, 13). A tool to quantify its extent could contribute to characterizing the individual pain phenotype, establishing an appropriate therapy, and following up on the disease's progress over time.

In the present study, we evaluated the feasibility of pressure pain testing in horses not exposed to this method before. Feasibility was assessed using a scoring system previously described for dogs (24) and adapted for the current experimental setup in horses. To maximize tolerability, horses were tested in their home stall under standard conditions. In 95% of subjects, it was easy to perform pressure testing and good to excellent cooperation was observed. This finding suggests better tolerability than initially expected. Indeed, we assumed that if 70% of the horses had tolerated the testing well, the method could have been considered clinically feasible. While this is an encouraging result, it might at least partially be linked to the particular population of horses included, as they had all been selected for military duties at the age of 3 years. These animals must be willing to work, agreeable and cooperative; therefore, they might not truly reflect the characteristics of a mixed population of sport and leisure horses commonly encountered in veterinary practice. For comparison, mechanical nociceptive testing using pressure algometry was feasible in 83% of dogs in the previously mentioned study (24) and in 83% of horses undergoing repeated testing over a period of 10 weeks (17).

In the present study, PPT was site-specific. In particular, P-PIP and D-DIP, on the proximal and distal interphalangeal joints, respectively, had lower thresholds than L-MCP, situated on the proximo-lateral aspect of the metacarpophalangeal joint. These results confirm previous observations, as different sensitivity to nociceptive mechanical stimulation for different anatomical areas has been previously reported in humans and horses. In humans, pressure sensitivity maps indicating distinctive sensitivity areas have been drawn for several body regions, such as the knee and the shoulder (14). In horses, higher thresholds were found in distal compared to proximal regions of the thoracic limb (17), in lumbar vs. thoracic back areas (19) and in specific sites of the face (29). As highlighted in previous literature, a direct comparison of PPT values among species and studies is only meaningful if the same instrument tip size, configuration and force application rate are applied (22, 30). Our thresholds are grossly comparable to those previously reported for distal equine thoracic limbs (average 5.2 N) (31) and donkeys (6.2 ± 2.1 N) (32), when, as in the present study, an algometer with a 2 mm diameter tip was applied. On the other side, they are largely different from those reported using a flat rubber tip of 1 cm^2^ (200 ± 40 N) (20). These differences highlight the importance of carefully considering technical details when comparing studies and results. Thus, for future clinical applications, in the absence of specific reference values for a specific equipment, stimulating tip and force rate increase, it seems reasonable to directly compare only threshold values found at the same joint and location bilaterally, rather than multiple sites on the same limb.

Our results concerning side differences confirm those of previous studies in equines indicating that no significant differences are present between PPTs measured on the left and right limbs for a given site (17, 20). This is an important finding, as consistent side differences in affected joints can be interpreted as possibly disease-related in clinical settings. In OA, both enhanced and decreased peripheral sensitization has been reported in other species, depending on the disease stage and age (13–16, 33). In humans, lower thresholds are typically reported for the affected joints compared to healthy controls (11) and similar results have been reported for horses undergoing experimentally induced carpal osteoarthritis (17).

As no significant differences were detected between sides, left and right threshold values for specific sites were averaged for further analysis. This approach has often been applied when establishing reference values for quantitative sensory testing methods in healthy subjects (17, 24). Similarly, it is common to perform multiple testing at specific sites and exclude extreme values from the averaging (16). As these tests are based on well-defined, objectively measurable inputs (i.e., the applied pressure) but on a merely behavioral output (i.e., the limb lifting or weight shifting), it is quite common to get outliers, as responses are unspecific. Keeping the two closest measured values and excluding one or two extremes enhance the probability of correctly defining thresholds (16). Thus, we followed this approach in the current study and recommend doing the same while applying the method in the clinical context.

In the current study, Freiberger horses, a traditional cold-type Swiss breed, and warmblood horses were enrolled. Anticipating that skin thickness differences might account for differences in thresholds, skinfolds at the scapula and neck basis were measured with a caliper. While no significant differences in pressure thresholds were found between breeds, thicker skin was found for Freiberger horses compared to warmblood. We assessed skinfold thickness using a caliper as commonly done in nutritional studies in humans. As the skin thickness was evaluated at proximal sites, it might reflect the presence of higher amount of subcutaneous fat in these body regions and thus not be adequate to estimate skin thickness at distal sites. For this purpose, ultrasound imaging at the site of interest would have been more adequate. To the best of our knowledge, this is the first study specifically assessing breed differences in pain sensitivity in horses. Future studies could investigate other modalities, such as thermal and tactile thresholds and other breeds, to substantiate this preliminary finding.

If a certain method is intended for use in clinical practice, it has to provide comparable results over time when applied to healthy individuals and when different observers perform the testing. Nevertheless, due to the semiquantitative nature of PPT, a perfect agreement between sessions and raters cannot be expected. To provide some examples from other species, when test-retest repeatability was evaluated, ICC ranging from 0.6 to 0.9 were reported in humans (11) and from 0.46 to 0.78 in horses, when the axial skeleton was evaluated with algometry, depending on the examiner (22, 34). Such ICC values were interpreted as showing adequate agreement in those studies. As it has been pointed out, several factors can influence repeatability, such as the body region to be tested, the experience of the examiner and the duration between sessions (22). In the present study, intra-rater reliability was evaluated by comparing thresholds obtained by a single observer in two testing sessions 2 weeks apart, measured at two sites on the same joint bilaterally. This testing paradigm was established to mimic a clinical situation in which a target joint would be tested on both limbs for internal comparison and retested at intermediate time intervals to verify the effect of treatment or disease progression. When averaged values per site were compared between sessions for the same observer, an agreement ranging from poor to good was found. For L-MCP, on the lateral aspect of the metacarpophalangeal joint, reliability was higher than for D-MCP, reflecting location-specific ease of testing in a repeatable way. This site-specificity is interesting to notice, as it might also be true for other joints. Sites located on the dorsal aspect of the limb might tend to hinder withdrawal more than lateral or palmar/plantar sites and thus originate higher variability in the results. This has been previously pointed out by Haussler in his review on algometry in horses (22), but further data would be necessary to confirm that a dorsal approach should rather be avoided when testing periarticular sensitivity. Furthermore, looking at threshold differences between sessions in the Bland-Altman plots it is evident that some individuals, mostly those having higher thresholds, were showing larger differences, reflected by the large range found for the 95% confidence interval of the intraclass correlation coefficient. Thus, horses having high initial thresholds might not be adequate candidate for a follow-up with algometry. Additionally, interesting to notice, is the absence of a clear direction of change over time, as some horses showed higher and other lower thresholds at the second appointment. In contrast, a previous study in horses found higher thresholds at the second appointment, with the extent of increase depending on the interval between sessions. A short interval of 1–3 days led to higher changes than 5–7 days (17). In dogs, opposite results were found, with mechanical thresholds decreasing at the second testing session (16, 35). This was interpreted as potential result of stress-induced analgesia at the first occasion (16, 35, 36), but it could also be explained by a learning process. Indeed, animals can learn that if they react earlier (i.e., at lower pressure), they might interrupt stimulation and thus avoid unpleasant feelings. In the author's experience, there are rather clear differences in the “true” evoked reactions and the learned early avoidance behavior, and this is at least partially reflected in the feasibility of testing. Animals that learn to react at minimal contact and do not “concentrate” on the testing should be excluded from this diagnostic modality. Thus, the number of repetitions, tested sites and the testing frequency might affect the results of mechanical nociceptive testing in animals. In Phases 2 and 3, we tested only two sites per limb, and the testing interval was 2 weeks. Shorter intervals and multiple sites or repetitions within a session have been shown to reduce tolerability (36).

When two raters performed the measurements, the agreement was good to excellent for both sites when tested on the same day at 1-h interval. This finding suggests that comparing pressure thresholds collected by different practitioners from the same horse over time would be acceptable.

Several devices have been described in the literature to measure mechanical nociceptive thresholds in horses. Most commonly, simple and cheap hand-held algometers with a 1 cm^2^ stimulating tip are used (22). The major drawback with these instruments is that the force or pressure application rate is not monitored, making repeatability inherently tricky. Indeed, in nociceptive testing, the force application rate strongly modulates the outcome. In the present study, a Prod Plus was used. This instrument, already described in other equine studies (21), was purposely developed for veterinary testing. It allows monitoring the force application rate through a practical LED light system and exchanging stimulating tips based on the species and site to be tested. As unpleasant pressure is finally responsible for evoking nocifensive responses, it is fundamental to apply force at a constant rate in a reliable way. The examiner manually exerts increasing force through the instrument, the applied pressure depending on the stimulating surface as clearly demonstrated for threshold determination in horses (30). Hence, the possibility of exchanging tips strongly increases testing reliability as the force necessary to evoke a response should remain acceptable for the operator. The cut-off force of 25 N suggested for this instrument guarantees the feasibility of testing for most operators and, on the other hand safety for the animals, as the risk of physical damage is minimized. Thus, the overall acceptable intra-rater and inter-rater reliability found in the present study is mainly linked to the adequacy of the instrument selected and to the stimulation protocol adopted.

We tested periarticular algometry in healthy horses to verify whether this method could become a complementary diagnostic tool for horses affected by OA. Importantly, great care should always be taken to avoid damage to the delicate anatomical structures that could arise from improper use of the testing instrument, including too fast force application rate, oblique positioning of the stimulating tip, or not respecting the recommended safety cut-off values. This careful approach should also and in particular apply whenever new bone formation and osteophytes are present or suspected, potentially modifying underlying anatomical structures and landmarks.

### 4.1 Limitations

The horses included in the present study were considered clinically healthy and free of lameness based on a thorough clinical examination, but not including imaging or laboratory testing. Only warmblood and Freiberger horses were included, thus the data presented here can only be considered representative for these two breeds. Evaluation of feasibility was performed merely for distal thoracic limb joints; no inferences can be made for pelvic limbs or proximal joints. Intra- and inter-rater reliability assessments were performed exclusively on data collected from two sites overlying the MCP joint and results might be different for other joints and sites.

## 5 Conclusion

Pressure pain mapping of distal thoracic limb joints was feasible in horses using the approach and equipment applied in the current study. Local sensitivity was site-specific and no side or breed differences were noticed. Data collected from the MCP joint suggest highly variable subject-dependent intra-rater reliability, ranging from poor to good, and good to excellent inter-rater reliability. Studies evaluating pathologic vs. healthy joints are needed before recommendations can be made about clinical usability and diagnostic validity.

## Data Availability

The raw data supporting the conclusions of this article will be made available by the authors, without undue reservation.
