# Supplementary material for: Pressure pain mapping of equine distal joints: feasibility and reliability
Source: Front Pain Res (Lausanne). 2024 Apr 25;5:1342954. doi: 10.3389/fpain.2024.1342954 (PMC11079115; doi:10.3389/fpain.2024.1342954)
Supplement: Supplementary file 1 [file Datasheet1.pdf]

APPENDIX 1 Health and lameness evaluation sheet

Horse identification

Date____________________ Day of Study____________________ Horse name_____________

Gender__________________ Age____________________________ Chip-Nr.________________

Breed____________________ Weight__________________________ BCS____________________

Physiological data

Heart rate_________________ Respiratory rate__________________ Rectal temperature_______

Digestive sounds____

Appearance	Bright, lowered head/ears, no reluctance to move	0
	Bright, alert, occasional head movements, no reluct. to move	1
	Restlessness, pricked ears, abnormal facial expr. pupils dilated	2
	Excited, continuous body movement, abnormal facial expr.	3
Sweating	No obvious signs of sweating	0
	Damp to the touch	1
	Wet to the touch, beads of sweat over entire body	2
	Excessive sweating, beads of water running off horse	3
Pawing on the floor	Quietly standing, no pawing	0
	Occasional pawing (1-2x per 5 min)	1
	Frequent pawing (3-4x per 5 min)	2
	Excessive pawing (>5x per 5 min)	3
Head movement	No evidence of discomfort, head straight	0
	Occasional head movement, laterally or vertically (1-2x in 5 min)<br>Lip curling (1-2x per 5 min), occasional look at flank	1
	Intermittent and rapid head movements (3-4x per 5 min)<br>Lip curling (3-4x per 5 min), frequent look at flank	2
	Continuous head movement, excessively looking at the flank<br>Lip curling (5x per 5 min)	3

Lameness evaluation

Adspection

Limbs (swelling, ect.):____

Limb conformation: ____

Hooves:____

Pulsation: ____

Degree of joint effusion	absent		mild		moderate		severe	
	L	R	L	R	L	R	L	R
Metacarpophalangeal joint:	0	0	1	1	2	2	3	3
Proximal interphalangeal joint:	0	0	1	1	2	2	3	3
Distal interphalangeal joint:	0	0	1	1	2	2	3	3

Reaction to joint palpation	absent		mild		moderate		severe	
	L	R	L	R	L	R	L	R
Metacarpophalangeal joint:	0	0	1	1	2	2	3	3
Proximal interphalangeal joint:	0	0	1	1	2	2	3	3
Distal interphalangeal joint:	0	0	1	1	2	2	3	3

Joint flexion	no response		mild		moderate		severe	
	L	R	L	R	L	R	L	R
Metacarpophalangeal joint:	0	0	1	1	2	2	3	3

Subjective lameness evaluation: *AAEP Lameness score*

Evaluation in hand, at walk and trot, on a straight line	Left	Right
Clinically sound	0	0
Inconsistently lame under certain circumstances	1	1
Consistently lame under certain circumstances	2	2
Consistently lame at the trot on the straight line	3	3
Lame at the walk	4	4
Non-weight bearing	5	5

Evaluation on the circle	Left circle		Right circle	
	L	R	L	R
Clinically sound	0	0	0	0
Mild intermittent lameness evident	1	1	1	1
Mild consistent lameness evident	2	2	2	2
Moderate lameness evident	3	3	3	3
Severe lameness evident	4	4	4	4
Non-weight-bearing	5	5	5	5

Neurological exam

Menace: ______________________________

Panniculus (lateral of the midline between shoulders to sacrum) ______________________________

Proprioception (crossing legs): ______________________________

Flexion tests (negative or positive), only in doubtful cases	Left	Right
Proximal joints	________	________
Carpus	________	________
Distal joints	________	________
Plank	________	________

Radiographic exams, only in doubtful cases

Joint, Findings:____

Joint, Findings:____

Feasibility score:

No problem	0
Mild difficulty	1
Moderate difficulty	2
Significant difficulty	3
Extreme difficulty	4
Impossible	5

Anti-inflammatory or other analgesic drugs:____

Last daily training exercise: (min 1h)____

Last shoeing date: ________________________________
